# Editorial: Linking Stomatal Development and Physiology: From Stomatal Models to Non-model Species and Crops

**DOI:** 10.3389/fpls.2021.743964

**Published:** 2021-09-30

**Authors:** Scott A. M. McAdam, Caspar C. C. Chater, Elena D. Shpak, Michael T. Raissig, Graham J. Dow

**Affiliations:** ^1^Purdue Center for Plant Biology, Department of Botany and Plant Pathology, Purdue University, West Lafayette, IN, United States; ^2^Royal Botanic Gardens, Kew, Richmond, United Kingdom; ^3^Department of Biochemistry, Cellular and Molecular Biology, University of Tennessee, Knoxville, Knoxville, TN, United States; ^4^Centre for Organismal Studies Heidelberg, Heidelberg University, Heidelberg, Germany; ^5^Molecular Plant Breeding, Institute of Agricultural Sciences, ETH Zurich, Zurich, Switzerland

**Keywords:** stomata/guard cells < gas exchange < physiology, guard cell development, stomatal evolution and behavior, plant abiotic response, stomatal ABA response, guard cell (GC), stomata

Stomata are highly dynamic valves in the epidermis of plants. These microscopic structures regulate the exchange of gases with the atmosphere and are essential for plant survival on land (Raven, 2002). There is an enduring fascination with stomata because of their specialized nature: from their unique development out of undifferentiated epidermal cells; to the environmental and internal signals they respond to; and the impacts their function have on climate and global change. These key themes have been the topic of many classical compendiums and scientific conferences (Jarvis and Mansfield, 1981; Ziegler et al., 1987; Roelfsema and Kollist, 2013). Research in the past two decades has accelerated our understanding of stomatal function, particularly through the accumulation of a critical mass of knowledge on the genetic underpinnings of stomatal development and physiology in the model angiosperm *Arabidopsis* (Assmann and Jegla, 2016; Qi and Torii, 2018). In this Frontiers eBook, we sought to bring together the latest research and reviews on stomatal biology that span a vast continuum: from cells to ecosystems. The articles were solicited with four key themes in mind: (1) The coordination of stomatal development with plant growth, development, and environmental signaling; (2) The role of stomatal development in plant acclimation and adaptation to the environment; (3) The influence of stomatal development and function on plant resource use, ecosystem processes, and global climate; and (4) The selection for stomatal traits in plant evolution, crop domestication and breeding, and designing food for the future.

The research contributed to this eBook encompasses the wide diversity of topics studied by contemporary stomatal biologists. At a phylogenetic level, works describe the unique stomata of mosses (Caine et al.; Renzaglia et al.), grasses (Buckley et al.; Serna), and a species of C_3_-CAM dicot *Mesembryanthemum* (Guan et al.). At the developmental level, articles describe critical stomatal developmental genes (Chen et al.), the physiological development of stomatal function in leaves (Kane et al.), and important signals for stomatal regulation in developing *Citrus* fruit (Lugassi et al.). At a functional scale, research spans the molecule to the leaf, with reports on the importance of aerosol deposition on stomatal function (Grantz et al.), the role of subsidiary cells in stomatal regulation (Gray et al.), the molecular link between reactive oxygen species (ROS) and abscisic acid (ABA) signaling in guard cells (Postiglione and Muday), and the importance of ROS and salicylic acid (SA) on stomatal responses to CO_2_ (He et al.). Novel and unexplored mechanisms are proposed by two papers, one which describes the evolutionary pressures placed on stomatal development by pathogens (Muir) and the other which finds divergent stomatal strategies driven by competition between species (Zenes et al.).

The bryophytes, including mosses, form a monophyletic group that is sister to all other land plants and represent the oldest extant lineage of plants to possess stomata, having diverged from the ancestor of vascular plants more than 400 million years ago (de Sousa et al., 2019). Caine et al. show shared and divergent control of stomatal ontogeny in the model moss species *Physcomitrium patens* through bHLH transcription factor and signaling peptide orthologs conserved in the angiosperm stomatal program (Chen et al.). In addition to the conserved one-cell spacing rule for moss stomata (Figure 1), the authors observe environmental plasticity in the development of moss substomatal cavities (Caine et al.), suggesting a more complex role for bryophyte stomata in the maturation of the reproductive sporophyte capsule. Insight into stomatal function in this lineage is scarce (Chater et al., 2016; Kubásek et al., 2021). In a comprehensive phylogenetic screening across mosses, Renzaglia et al. find that stomata have been lost more than 63 times across this lineage and there is considerable variation in the number of stomata per capsule within and between families. This discovery raises questions about the possible functions of stomata in bryophytes, and by extension in the common ancestor of all extant land plants. Renzaglia et al. suggest that stomata are functionally dispensable for spore dispersal in mosses, although their continued presence in species that do have stomata suggests that stomatal opening offers a fitness advantage by facilitating the desiccation of the sporophyte capsule (Caine et al.).

**Figure 1:**
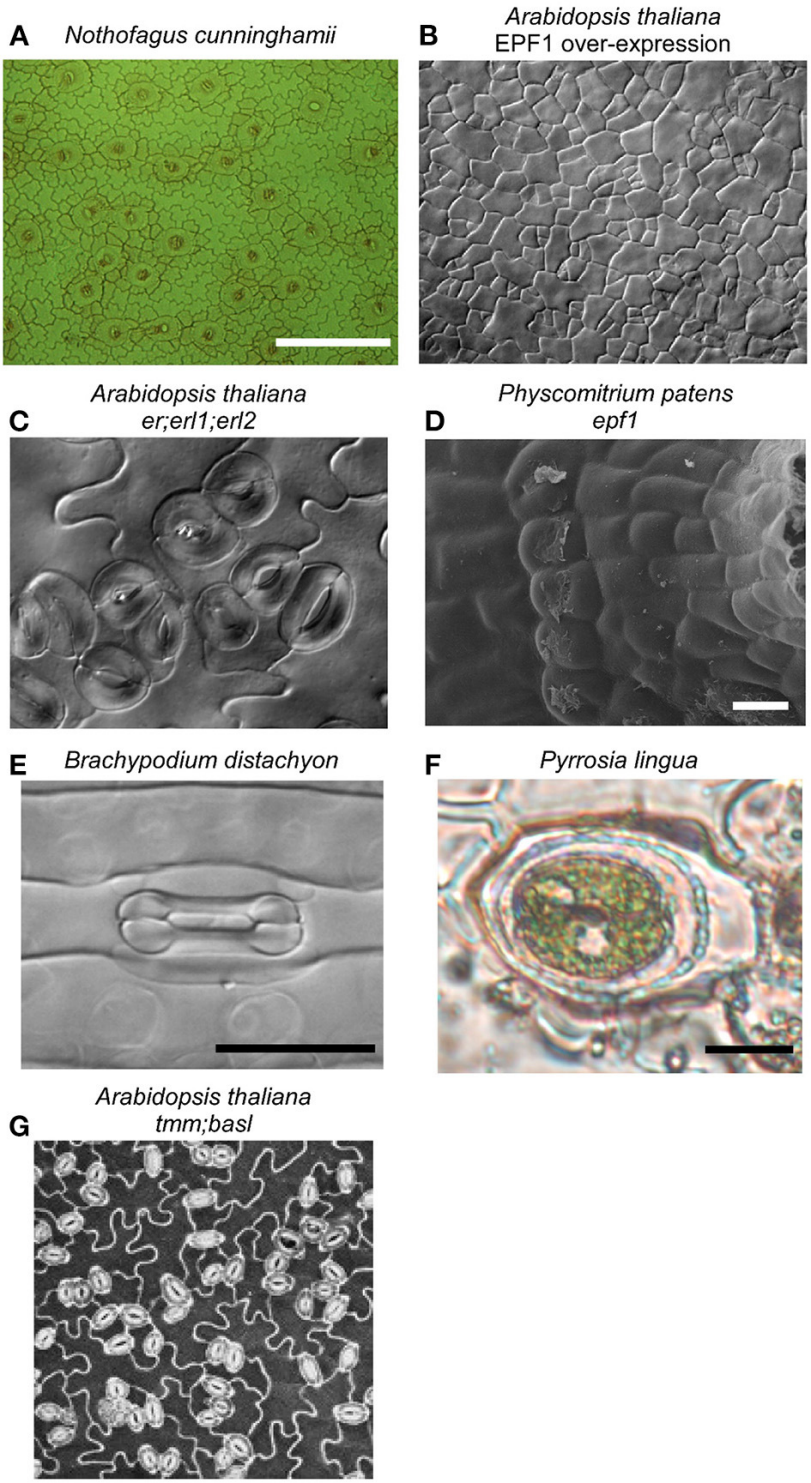
Stomata are found on the aerial parts of most land plants, in the highest densities on the leaves of vascular plants, like the temperate rainforest tree *Nothofagus cunninghamii* **(A)**. Considerable insights into the molecular determinants of stomatal development have been made in *Arabidopsis thaliana*, with the discovery that SPEECHLESS is essential for stomata formation; when it is constitutively inhibited by the EPF/ERF signaling pathway, epidermal cells can not differentiate into stomata **(B)**. Mutants in this pathway, like the *er;erl1;erl2* triple mutant in *Arabidopsis* **(C)** and *epf1* mutant in the moss *Physcomitrium patens* (Caine et al., 2016) **(D)**, both having similarly clustered stomata, indicating that all land plants use the same pathway for stomatal development. Considerable diversity in stomatal form exists across land plants, exemplified by the dumbbell-shaped guard cells and associated subsidiary cells of some species of monocot, including the grass *Brachypodium distachyon* **(E)**. Subsidiary cell arrangement is highly diverse across land plants, in some ferns including *Pyrrosia lingua* both guard cells are surrounded by a single, encircling subsidiary cell **(F)**. Neighboring cells can impact stomatal function, with compromised stomatal responses reported in stomatal developmental mutant lines that have increased stomatal clustering (Dow et al., 2014), like the *tmm;basl* double mutant of *Arabidopsis* **(G)**.

Grasses, of which domesticated cereal crops provide more than 50% of globally consumed calories (Yu and Tian, 2018), have long been recognized as having unique stomatal complexes comprised of dumbbell-shaped guard cells flanked by highly specialized subsidiary cells (Strasburger, 1866; Figure 1). This stomatal complex may have evolved to enhance stomatal response speed, and is only found in Poaceae and Cyperaceae (Raschke, 1975; Nunes et al., 2020). Only recently are we starting to reveal the genes responsible for the development of these unique stomatal complexes. Serna provides a contemporary perspective on our understanding of the genetic control of C_3_ and C_4_ grass stomatal complexes, in which orthologs of the bHLH transcription factor MUTE have been co-opted as regulators of subsidiary cell differentiation and specialization (Raissig et al., 2016; Wang et al., 2019; Wu et al., 2019). Buckley et al. provide a highly complementary review to this discussion on the unique developmental regulators of grass stomata, by detailing the possible gene targets in the stomatal development network that could be utilized for the generation of climate-ready cereal crops. The genetic regulation of stomatal development in grasses stands in contrast to the regulation of stomatal development in other species (Chen et al.), for example, provide a compelling case for the pivotal importance of the bHLH transcription factor SPEECHLESS in the development of stomata in *Arabidopsis* (Figure 1).

The grasses provide an extreme example of the subsidiary cells that flank guard cells (Figure 1). There are numerous unique subsidiary cell arrangements described across land plants (Baranova, 1987), yet their functional role and relevance remain mostly enigmatic. Gray et al. provide a modern synthesis on subsidiary cell development, diversity, and what little we know about subsidiary cell function. Gray et al. conclude that future work utilizing new model systems with highly complex subsidiary cell arrangements has the potential to advance our understanding of how they develop and their roles in stomatal function across diverse plants. It is tempting to speculate that subsidiary cell morphological complexity is indicative of a broad functional diversity as extensions of the stomatal complex; perhaps providing spatio-temporally distinct reservoirs of essential metabolites, ions, and signaling components for optimal guard cell responses or mechanically accommodating guard cell movement. Future single-cell transcriptomic and metabolomic studies may help determine exactly what these subsidiary cell functions are.

For over 150 years researchers have investigated the environmental and endogenous signals that cause stomata to open and close in mature tissue (von Mohl, 1856), however very little work has focused on how stomatal function develops (Pantin et al., 2013). To address this, Kane et al. investigated when stomata begin to function in developing leaves. Newly expanding leaves tend to have high rates of water loss and the authors show the majority originates from the immature cuticle and not stomata. In young leaves, stomata have not yet fully developed and remain shut; they only appear to open and control water loss once the leaf is approaching full expansion and cuticular conductance reaches a minimum (Kane et al.). Stomata are not only found on leaves; in many angiosperm species stomata are also found on the epidermis of fruit (Blanke and Lenz, 1989). Lugassi et al. investigated stomatal function as fruit develop and found that stomata on immature *Citrus* fruit are functional and responsive to sugars via hexokinase, just like a leaf. As fruit develop, stomata become plugged, which greatly reduces fruit transpiration. Lugassi et al. developed a transgenic *Citrus* plant that had guard cell specific expression of hexokinase and found that these plants had reduced seed development and more closed stomata during fruit formation, suggesting that functional stomata on immature *Citrus* fruit are critical for seed formation. Physiologically active stomata on green immature fruits likely contribute a carbon source for photosynthesis in this rapidly developing organ, prior to their switch from source to carbon sink duration maturation; correlating with the loss of the fruit's stomatal function and photosynthetic capacity. The timing of this transition could be a breeding target for the control of fruit ripening and flavor traits.

Since the advent of stomatal research, responses have been detected via direct measurement of single pores or gas exchange measurements providing mean approximations of the aperture of many thousands of stomata (Darwin, 1898). Grantz et al. show that while there is considerable variation in individual stomatal apertures across a large leaf surface area exposed to similar conditions, stomatal apertures are quasi-normally distributed, and remain so when responding to VPD and atmospheric pollutants. This result provides an important experimental bridge between measurements of stomatal responses made at a local, individual pore scale, with those measured at a leaf level (Grantz et al.).

Stomatal closure is a key adaptation to conserve water during periods of water deficit or to optimize water use relative to photosynthetic carbon gain (Cowan and Farquhar, 1977; Brodribb et al., 2017). Characterizing the signals of stomatal closure is a core target of modern stomatal biology and has great potential for increasing crop productivity via modern breeding techniques (Anjanappa and Gruissem, 2021; Horton et al., 2021). Halophytes, or plants that can survive in high salt environments, have evolved some of the most extreme adaptations to retain and use scarce available water, including adaptations to stomatal regulation (Shabala, 2013). *Mesembryanthemum crystallinum* is an emerging model system for discovering the molecular pathways of plant tolerance to salt or extremely limited available free-water (Guan et al.). Guan et al. characterized the unique ability of this plant to switch between C_3_ and CAM photosynthesis under salt stress, finding the transition takes approximately 6 days and elicits a major switch in diurnal stomatal rhythm and photosynthetic regulation. The hormone abscisic acid (ABA) plays a critical role in stomatal closure during water deficit and the signaling pathway for this direct response is well described (Geiger et al., 2010; Brandt et al., 2012). Postiglione and Muday provide a thorough review of the role of ABA-induced ROS signaling in guard cell regulation, a signaling pathway that operates in parallel to the direct ABA pathway. Postiglione and Muday conclude that ROS are a critical component of guard cell ABA signaling and that future work will reveal the relative importance of the direct action of ABA and ROS on stomatal responses. In parallel to this work, He et al. demonstrate that both ROS and salicylic acid (SA) are essential in the stomatal response to elevated CO_2_ in *Arabidopsis*. He et al. found that ROS originates from two different sources in the guard cells depending on whether stomata are closed by elevated CO_2_ or if light-induced opening is inhibited by it. Furthermore, SA is required for elevated CO_2_-induced stomatal closure (He et al.), increasing the complexity of the hormonal landscape of guard cell regulation (Jarvis and Mansfield, 1981).

Modeling continues to be a powerful tool to propose and test hypotheses related to stomatal function and development (Ziegler et al., 1987). To this end, Muir developed a new model that examines the trade-off between maximum anatomical stomatal conductance, as determined by stomatal size and density, and the risk of pathogen infection. This model proposes some non-intuitive, yet testable, hypotheses related to the evolution of stomatal development in response to pathogen infection risk and provides a new framework for understanding the developmental trade-offs between maximizing leaf gas exchange and minimizing infection (Muir). Zenes et al. also utilize a new model of plant water use to analyze experimental findings on the shift in plant water use strategies in response to competition. The adaptation in water use strategy in response to competitors can be accurately predicted by a model that assumes plants maximize carbon gain while only mitigating water use that prevents the risk of hydraulic failure by embolism (Zenes et al.). This result shifts our understanding of how plants manage water use away from exclusively optimizing carbon gain relative to water use, toward maximizing carbon gain while preventing lethal hydraulic thresholds (Anderegg et al., 2018). The mechanism behind these shifts in water use strategy in response to competition remain uncharacterized and provides an exciting prospect for future studies. Although (Zenes et al.) focus on C3 tree seedlings, it would be interesting to see how this model fairs with C4 and CAM plant species.

## Conclusion

Modern stomatal biology continues to investigate research questions that span a vast continuum of disciplines from the cell to the ecosystem. In this Research Topic “Linking Stomatal Development and Physiology: From Stomatal Models to Non-models and Crops,” research articles and reviews cover much of this continuum. Among the topics included: ancient evolution of stomatal development and function; further description of the complex genetic models governing stomatal development; and the identification of breeding targets for improved water use and productivity in agricultural systems. Nevertheless, with such a broad topic as stomata, many themes inevitably remain unaddressed. For example, insights into C_2_ and C_4_ stomatal responses, circadian stomatal rhythms, and stomatal thermal responses are just some of the areas not covered here. However, a universal theme amongst this diverse stomatal research, linking plant gas exchange and photosynthetic mechanisms, is testament to the sheer importance that these tiny plant structures have on our understanding of plant development, function, and evolution.

## Author Contributions

SM wrote the first draft and all co-authors contributed equally to writing the manuscript and developing the figure. All authors contributed to the article and approved the submitted version.

## Conflict of Interest

The authors declare that the research was conducted in the absence of any commercial or financial relationships that could be construed as a potential conflict of interest.

## Publisher's Note

All claims expressed in this article are solely those of the authors and do not necessarily represent those of their affiliated organizations, or those of the publisher, the editors and the reviewers. Any product that may be evaluated in this article, or claim that may be made by its manufacturer, is not guaranteed or endorsed by the publisher.
